# Electroacupuncture Ameliorates Alcohol‐Related Cognitive Impairment by Modulating the Gut‐Brain Axis to Alleviate LPS Driven Neuroinflammation

**DOI:** 10.1002/cns.71213

**Published:** 2026-10-16

**Authors:** Yueqin Liu, Jianjie Luo, Shuyou Wang, Yinwen Wu, Shuyong Jia, Guangjun Wang, Weiguo Xue, Xiaojing Song

**Affiliations:** ^1^ Medical Engineering Laboratory, Institute of Acupuncture and Moxibustion China Academy of Chinese Medical Sciences Beijing China; ^2^ School of Acupuncture‐Moxibustion and Tuina Beijing University of Chinese Medicine Beijing China

**Keywords:** alcohol‐related cognitive impairment, electroacupuncture, gut‐brain axis, lipopolysaccharide, neuroinflammation

## Abstract

**Objective:**

To investigate whether electroacupuncture (EA) at Zusanli (ST36) ameliorates alcohol‐related cognitive impairment (ARCI) by restoring intestinal barrier integrity and to elucidate the role of the vagus nerve in this “gut‐to‐brain” protective mechanism.

**Methods:**

An ARCI mouse model was established by daily ethanol gavage for 28 days. Mice received EA at ST36 from day 15 onwards. Behavioral tests (MWM, OFT, EPM) were conducted to assess cognitive and emotional function. Time‐course analysis of intestinal pathology (H&E, ZO‐1), permeability (FITC‐LPS, DAO), and neuroinflammation (LPS, IBA‐1, cytokines) was performed. The role of the vagus nerve was investigated using ipsilateral DMV lesion and exogenous LPS challenge.

**Results:**

Chronic alcohol exposure caused significant cognitive deficits, anxiety‐like behaviors, and body weight loss, accompanied by early‐onset intestinal barrier disruption and elevated LPS, which preceded central neuroinflammation. EA at ST36 significantly ameliorated these behavioral and pathological changes. EA activated DMV cholinergic neurons, restored ZO‐1 expression, and reduced LPS translocation. Crucially, both DMV lesion and exogenous LPS administration abolished EA's protective effects on the intestinal barrier, neuroinflammation, and behavior.

**Conclusion:**

EA at ST36 alleviates ARCI by activating the vagus nerve to restore intestinal barrier integrity, thereby preventing gut‐derived LPS from triggering neuroinflammation. This identifies a key vagal‐dependent mechanism and supports EA as a promising intervention for ARCI.

## Graphical abstract

Electroacupuncture at ST36 can ameliorate alcohol‐related cognitive impairment by modulating the gut‐brain axis to alleviate LPS driven neuroinflammation. It is a potential strategy for preventing and treating ARCI.

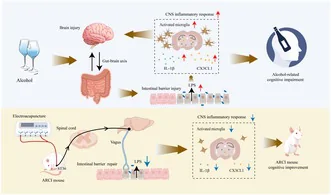

## 1 Introduction

Alcohol‐related brain damage (ARBD) is an important global cause of neurocognitive disorders [1, 2]. Its most common early manifestation, alcohol‐related cognitive impairment (ARCI), affects up to 80% of patients with alcohol use disorder [3, 4]. Clinically, ARCI presents as progressive deficits in memory, executive function, emotional regulation, and without timely intervention, often progresses to irreversible dementia [5]. Despite its high prevalence, there are currently no generally recognized effective interventions for ARCI. Existing management strategies primarily focus on alleviating alcohol withdrawal symptoms and providing symptomatic treatment, with commonly used drugs including disulfiram and naltrexone [6, 7]. However, these approaches have limited efficacy and are associated with significant side effects. Consequently, there is an urgent need for safe, effective, mechanism‐based interventions for ARCI.

The gut‐brain axis is a key interventional target for neuroinflammation and cognitive impairment, and intestinal barrier dysfunction is recognized as a significant driver of central nervous system (CNS) patholgy [8, 9]. Alcohol exposure induces pathological changes in intestinal structure, including villus damage [10, 11, 12], and promotes the development of a “leaky gut” [13, 14]. This allows lipopolysaccharide (LPS) to enter first the circulation, and then the brain, leading to the activation of hippocampal microglia, the primary immune effector cells of the CNS [15, 16]. Overactivated microglia exhibit a proinflammatory phenotype [17], releasing interleukin‐1 beta (IL‐1β), CX3 chemokine ligand 1 (CX3CL1), and reactive substances. These factors subsequently damage synapses, inhibit neurogenesis, and impair hippocampal function [18, 19], ultimately leading to the cognitive decline observed in ARCI patients. Thus, regulating the gut‐brain axis may be an effective approach for the prevention and treatment of ARCI.

Acupuncture has been recognized by the World Health Organization (WHO) as a safe intervention for alcohol‐related disorders [20]. Importantly, acupuncture therapy has shown significant efficacy in treating patients with alcohol addiction and intoxication [21, 22, 23]. Studies have demonstrated that acupuncture can improve intestinal barrier function, thereby modulating the gut‐brain axis [24, 25]. Specifically, electroacupuncture (EA) stimulation at Zusanli (ST36) shows potential in reducing neuroinflammation and cognitive deficits in animals [26, 27, 28]. However, whether EA at ST36 has a therapeutic effect on the cognitive impairment that occurs in the early stage of ARBD? Whether this brain protective effect relates to regulating the gut‐brain axis? This is a topic worthy of further research.

Advancing translational research on ARCI requires mechanistic studies that connect with clinical applications. In this study, we integrated behavioral, intestinal barrier, neuroinflammatory, and vagal endpoints, aiming to provide evidence relating to how EA influences the gut‐brain axis in ARCI, as well as data to support the use of EA as a translatable intervention for this condition. Our findings are expected to provide strategies for slowing the progression of ARCI and fill a critical gap in clinical practice relating to this disease.

## 2 Materials and Methods

### 2.1 Animals and Group Allocation

Four week old male Kunming mice, weighing 18~20 g, were obtained from Biotechnology Co. Ltd. (Beijing, China). The experimental procedures were in accordance with the National Institutes of Health Guide for the Care and Use of Laboratory Animals. The mice were housed in standard cages at 24°C ± 2°C under a 12 h/12 h light/dark cycle with free access to food and water for at least 1 week before the start of the experiments.

### 2.2 ARCI Model Establishment

The mice were given saline or 50% ethanol (15 mL/kg body weight) by gavage daily for 28 days as previously described [29]. All mice were randomly allocated into three groups: a control (Ctrl) group (*n* = 10), which was administered saline; an ARCI model (Alc) group (*n* = 13), administered 50% ethanol; and an EA group (*n* = 12), in which the animals were given 50% ethanol and then treated with EA. Because alcohol gavage can be lethal to mice, a greater number of mice were allocated to the Alc and EA groups than to the Ctrl group. During the study, three mice in the Alc and two in the EA group died.

### 2.3 EA Intervention

Mice in the EA group received EA intervention from day 15 post gavage, lasting for 14 days. The ST36 acupoint, located on the posterolateral side of each knee approximately 2 mm below the fibular head, was selected as the intervention site. A pair of stainless steel needles (0.16 × 7 mm) was inserted to a depth of 2–3 mm at bilateral ST36 acupoints on the hind limbs. Subsequently, EA was applied to both hind limbs using an electroacupuncture apparatus (Model SDZ‐III, Suzhou, China). A dense‐disperse wave was used at alternating frequencies of 2 Hz and 50 Hz with a current intensity of 1 mA, until slight and regular trembling of the bilateral hind limbs was observed. Each EA session lasted 20 min, once daily for 14 days. All EA procedures were performed by the same trained researcher to minimize inter‐operator variability.

### 2.4 Additional Intervention Groups for Mechanistic Study

To investigate the mechanisms underlying the effects of EA, two additional groups were included: the EA + i.DMV group and the EA + LPS group.

For the EA + i.DMV group, mice were anesthetized with 3% isoflurane and fixed on a stereotaxic frame (RWD 68025, RWD Life Science, China). A 26‐gauge inner cannula (protruding 1.0 mm below the guide cannula) was connected to a 1.0 μL Hamilton syringe. Sustained‐release muscimol (0.5 μL, 0.075–0.10 μg) was microinjected into the right DMV (AP: −7.32 mm, ML: −0.40 mm, DV: −5.20 mm from bregma; injection site verified by cresyl violet staining in preliminary experiments) at a flow rate of 100 nL/min. The cannula was left in place for 60 s after injection to ensure adequate diffusion. EA was then performed according to the protocol described in Section 2.3.

For the EA + LPS group, mice received EA combined with intraperitoneal injection of LPS (3 mg/kg) administered 30 min before each EA session, from day 15 to day 28.

All mice in these additional groups underwent the same behavioral tests and sample collection procedures as described for the EA group.

### 2.5 Behavioral Tests

Behavioral tests were sequentially performed from day 24 to day 30 after ethanol gavage, including the Morris water maze (MWM) test, open field test (OFT), and elevated plus maze (EPM) test. The MWM test was conducted from day 24 to day 28, consisting of 4 days of place navigation trials (four 60‐s sessions per day), and a spatial probe test on the final day; EthoVision XT (Noldus Information Technology, The Netherlands) was used to track and record the escape latency, distance traveled, and locomotor trajectories, to perform cognitive scoring of the adopted search strategies based on these trajectories, as well as to record platform crossings and percentage of distance in the target quadrant [30, 31]. On day 29, the OFT was carried out in a black open field box (40 × 40 × 40 cm) under sufficient illumination; each mouse was individually placed in the center of the box and allowed to freely explore for 5 min, with EthoVision XT recording the total distance traveled and time spent in the central area. The EPM test was performed 1 day after the OFT: each mouse was placed at the center of the EPM for 5 min, and EthoVision XT recorded the number of entries into the open arms and the time spent in the closed arms.

### 2.6 In Vivo Fluorescence Imaging (IVFI)

Mice were administered FITC‐LPS (Sigma‐Aldrich, Cat. No. F3665, *Escherichia coli* O111:B4, 50 μg/mL, 0.2 mL per mouse) via enema. Six hours after the enema, mice were sacrificed, and brain tissues were rapidly dissected and harvested. Fluorescence imaging was conducted using an FX PRO multimodal in vivo imaging system (Carestream Health Inc., Woodbridge, CT, USA). The fluorescence intensities in the brain were analyzed using Carestream molecular imaging software (Carestream Health Inc., Rochester, NY, USA).

### 2.7 Probe‐Based Confocal Laser Endomicroscopy (pCLE)

After anesthesia, the mid segments of the jejunum were surgically excised from mice, longitudinally incised, and immersed in 1% sodium fluorescein for 2–3 min. A probe‐based confocal laser endomicroscope (ViewnVivo, Australia) was then placed on the jejunal mucosa to image the structure of the mucosal layer (scanning depth: 14 μm; laser power: 20 μW). Three or four clear fields of view were randomly acquired per mouse.

### 2.8 Enzyme Linked Immunosorbent Assay (ELISA)

The levels of LPS in mouse serum and brain tissue samples were determined using a high‐sensitivity LPS ELISA kit (Solarbio, Cat# SEKSM‐0066), while diamine oxidase (DAO) levels in the serum of the same mice were measured using a Mouse DAO ELISA Kit (Solarbio, Cat# SEKM‐0398). For LPS detection, undiluted brain tissue homogenates and 10‐fold diluted serum samples were used; for DAO detection, all samples were diluted 10‐fold. All experiments were performed strictly in accordance with the manufacturer's operating instructions.

### 2.9 Histological Staining

Brain tissues were dehydrated in 30% sucrose and sectioned into 30‐μm frozen sections for immunofluorescence. Jejunal tissues were processed into 5‐μm paraffin sections; some were used for immunofluorescence, and others were used for hematoxylin and eosin (H&E) staining. After blocking, the sections for immunofluorescence were incubated with primary antibodies at 4°C overnight, followed by corresponding secondary antibodies in the dark. All immunofluorescence sections were mounted with DAPI‐containing antifade medium. Frozen brain sections were observed under a laser confocal microscope (FV1200, Olympus), and the immunofluorescence‐stained paraffin jejunal sections were examined under a fluorescence microscope (ECLIPSE C1, Nikon). Jejunal sections for H&E staining were stained using standard protocols and imaged with an Olympus BX53 microscope. Representative micrographs were obtained from different independent animals in each experimental group. Detailed antibody information is listed in Table S1 of the Supporting Information.

### 2.10 Statistical Analysis

Statistical analyses were performed with SPSS 22.0 (IBM, USA), and GraphPad Prism 9.5 (San Diego, USA) was used for graphing. All data were tested for normality and homogeneity of variance. One‐way ANOVA and two‐way repeated‐measures (RM) ANOVA were used for statistical analysis. All multiple comparisons were performed using the Bonferroni post hoc test. All results are expressed as means ± standard error of the mean (SEM). A *p* value < 0.05 was considered statistically significant.

## 3 Results

### 3.1 EA at ST36 Ameliorates Physical Parameters in Chronic Alcohol‐Exposed Mice

The experimental schedule for EA intervention in the ARCI mouse model is illustrated in Figure 1A. As shown in Figure 1B, mice in the Ctrl exhibited lustrous fur, a robust physique, strong activity, and good overall condition. In contrast, animals in the Alc were emaciated, displayed disheveled and dull fur, and showed irritability with reduced activity. Notably, these adverse effects were markedly suppressed following EA intervention.

**FIGURE 1:**
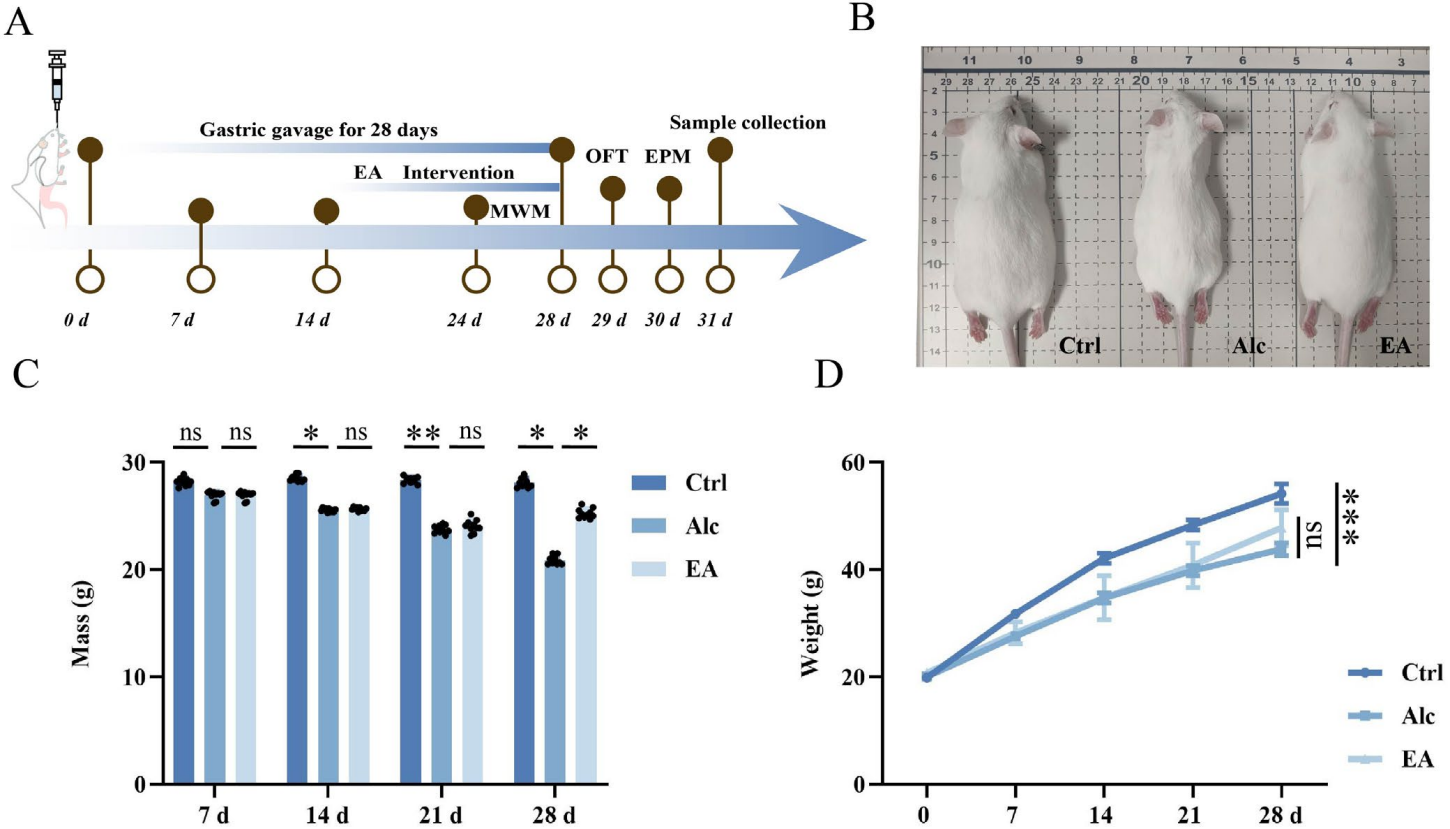
EA at ST36 ameliorates alcohol‐induced deterioration of general physiological parameters. (A) Schematic diagram of the experimental timeline. Ctrl, control group; Alc, alcohol‐fed group; EA, alcohol‐fed group treated with EA at ST36. (B) Representative images of mice from each group at the end of the experimental period. (C) Weekly average food intake. (D) Weekly body weight changes. *p < 0.05; **p < 0.01; ***p < 0.001; ns, *p* > 0.05. Weekly average food intake was analyzed by one‐way ANOVA, and body weight was analyzed by two‐way repeated measures ANOVA. All multiple comparisons were conducted with the Bonferroni post hoc test. *n* = 10 per group. All data are expressed as mean ± SEM.

At the initiation of the study, food intake was comparable among the three groups. Food intake in the Alc was significantly decreased on days 14 (*p* < 0.05), 21 (*p* < 0.01), and 28 (*p* < 0.05), compared to the Ctrl. Interestingly, after 14 days of EA intervention (day 28 post‐modeling), ARCI mice displayed a significant improvement in food intake (*p* < 0.05, Figure 1C). Similarly, body weight in the Alc was lower relative to the Ctrl on day 28 post‐exposure (*p* < 0.001), a trend that was partially reversed following EA intervention (Figure 1D).

### 3.2 EA at ST36 Attenuates Chronic Alcohol Exposure‐Induced Learning and Memory Impairment in Mice

In the MWM test (Figure 2A), mice underwent 1 day of adaptation training followed by 4 days of place navigation trials, with a spatial probe test conducted on the final day. The swimming trajectories of mice in the three groups on the last day of the place navigation trials were recorded (Figure 2B). Over the place navigation trials period, the average latency to find the platform decreased in all the groups; however, the decrease in escape latency in mice of the Alc was minimal, and significantly differed from that of the other two groups (*p* < 0.001, Figure 2C). On the last day, we analyzed the distance traveled among the three groups. We found that mice in the Alc swam a markedly longer distance to locate the escape platform than those in the Ctrl (*p* < 0.05, Figure 2D), indicating that chronic alcohol exposure led to impaired learning and memory. Notably, the distance to locate the escape platform was substantially reduced in the EA relative to that in the Alc (*p* < 0.001, Figure 2D). In agreement with these findings, the average cognitive score was significantly lower in the Alc than in both the Ctrl (*p* < 0.001, Figure 2E) and EA (*p* < 0.01, Figure 2E).

**FIGURE 2:**
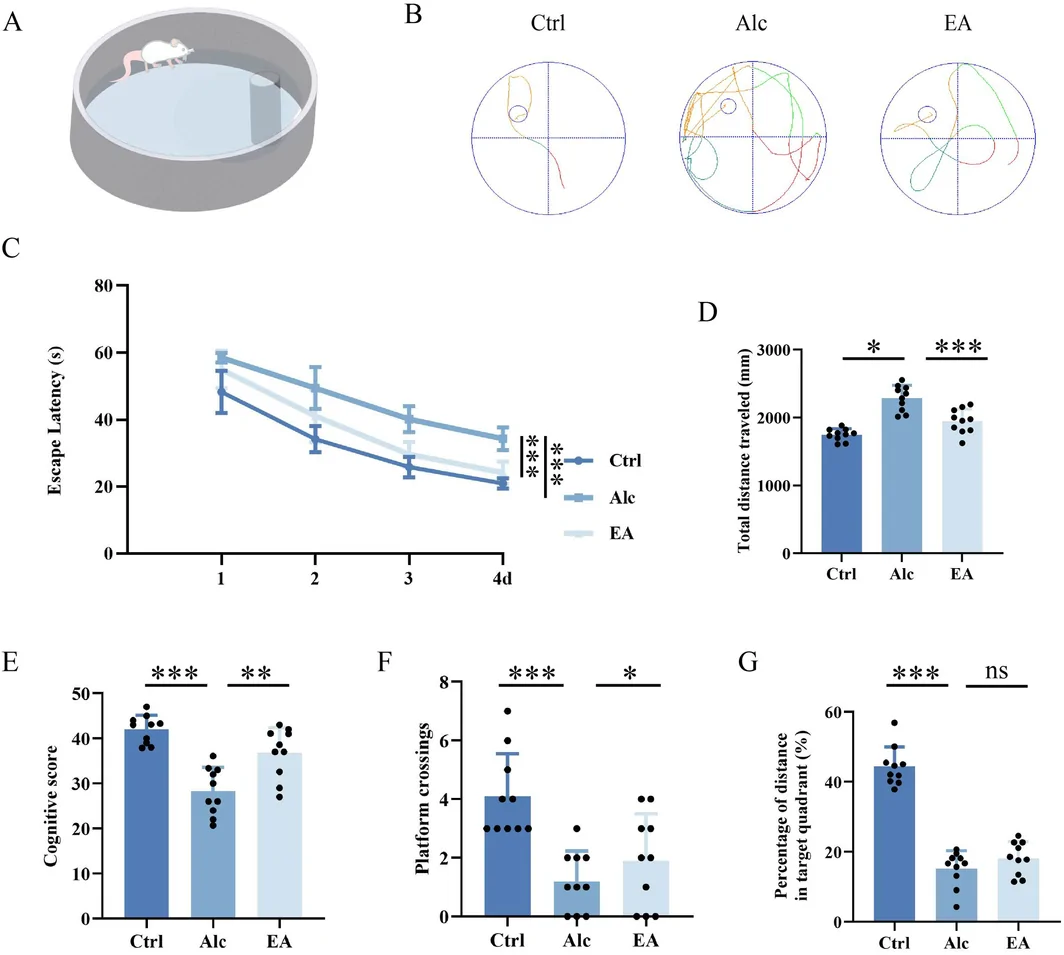
EA at ST36 alleviates cognitive deficits induced by chronic alcohol exposure. (A) Schematic diagram of the Morris water maze (MWM) test. (B) Representative swimming trajectories during the spatial probe test, Ctrl, control group; Alc, alcohol‐fed group; EA, alcohol‐fed group treated with EA at ST36. (C) Escape latency during the 5‐day place navigation test. (D) Total distance traveled to reach the hidden platform on the final day of place navigation. (E) Cognitive score determined by platform‐based search strategy analysis. (F) Number of platform crossings during the spatial probe test. (G) Percentage of time spent in the target quadrant during the spatial probe test. *p < 0.05; **p < 0.01; ***p < 0.001; ns, *p* > 0.05. Escape latency was analyzed by two‐way repeated measures ANOVA, and other data were analyzed by one‐way ANOVA. Multiple comparisons were performed using the Bonferroni post hoc test. *n* = 10 per group. Data are presented as mean ± SEM.

Furthermore, in the spatial probe test, compared with the Ctrl, mice in the Alc showed a marked decrease in the number of times crossing the former platform location (*p* < 0.001, Figure 2F) and time spent in the target quadrant (*p* < 0.001, Figure 2G), indicating that spatial recognition memory was impaired in these mice. Meanwhile, EA resulted in an increase in the number of platform crossings (*p* < 0.05, Figure 2F) and the time spent in the target quadrant, suggesting that this therapy mitigated the recognition memory impairment induced by chronic alcohol exposure.

### 3.3 EA at ST36 Reduces Anxiety and Exploratory Behavior in Chronic Alcohol‐Exposed Mice

The open field test (OFT; Figure 3A) and the elevated plus maze (EPM; Figure 3E) were employed to assess anxiety and exploratory behavior. Representative trajectories from the three groups in the OFT and EPM are shown in Figure 3B,F, respectively. In the OFT, mice in the Alc traveled shorter total distances (*p* < 0.001, Figure 3C) and spent less time in the central area (*p* < 0.05, Figure 3D) than those in the Ctrl group. Similarly, in the EPM test, Alc mice made fewer entries into the open arms (*p* < 0.01, Figure 3G) and spent more time in the closed arms (*p* < 0.05, Figure 3H) compared to their control counterparts. These observations indicate that chronic alcohol exposure led to heightened anxiety‐ and depression‐like behaviors in mice. Conversely, mice in the EA traveled longer total distances (*p* < 0.01, Figure 3C) and spent more time in the central area than Alc mice in the OFT (*p* < 0.05, Figure 3D). Additionally, EA intervention also increased the number of entries into the open arms by model mice and reduced the time they spent in the closed arms (*p* < 0.05, Figure 3G,H). These results reveal that EA has the potential to modulate anxiety‐ and depression‐like behaviors.

**FIGURE 3:**
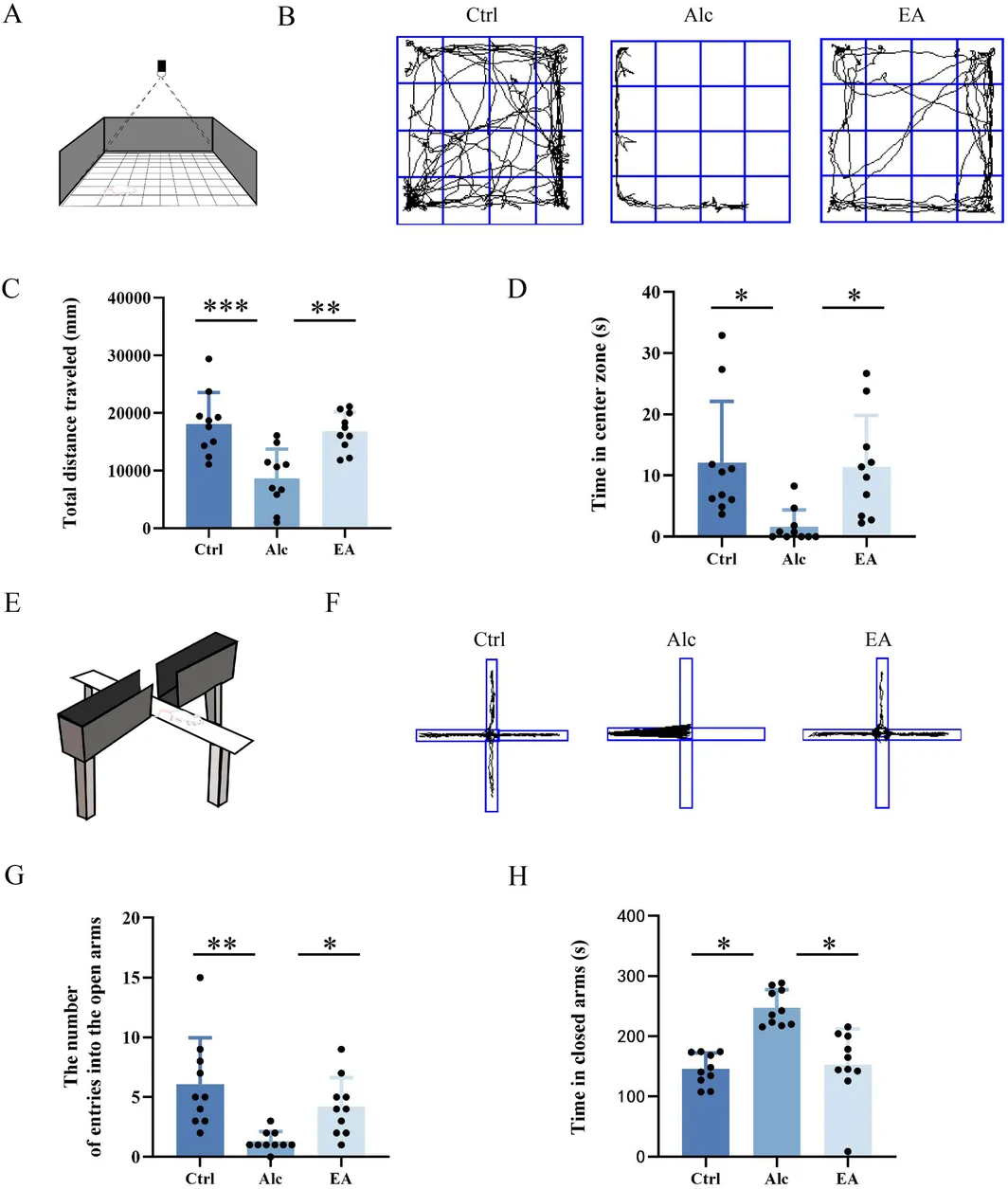
EA at ST36 attenuates anxiety‐like behaviors in alcohol‐exposed mice. (A) Schematic diagram of the open field test (OFT). (B) Representative movement trajectories in the OFT, Ctrl, control group; Alc, alcohol‐fed group; EA, alcohol‐fed group treated with EA at ST36. (C) Total distance traveled in the OFT. (D) Time spent in the center zone of the OFT. (E) Schematic diagram of the elevated plus maze (EPM) test. (F) Representative movement trajectories in the EPM. (G) The number of entries into the open arms of the EPM. (H) Time spent in the closed arms of the EPM. *p < 0.05, **p < 0.01, ***p < 0.001. Data were analyzed by one‐way ANOVA. Multiple comparisons were performed using the Bonferroni post hoc comparisons. *n* = 10 per group. Data were presented as mean ± SEM.

### 3.4 Intestinal Barrier Injury Precedes Central Neuroinflammation in Chronic Alcohol‐Exposed Mice

To establish the temporal sequence between intestinal pathology and central inflammation, we analyzed a time course of alcohol exposure (W0–W4; Figure 4). Histological examination revealed progressive jejunal villus damage beginning as early as week 1, characterized by structural injury at villus tips and mild swelling on H&E staining. This pathological damage progressively worsened with prolonged alcohol exposure, reaching peak severity at week 4 (Figure 4A). Immunofluorescence staining confirmed that intestinal tight junction protein ZO‐1 expression was significantly decreased from week 1 and exhibited a progressive downward trend throughout the modeling period (*p* < 0.001 vs. W0, Figure 4B,C).

**FIGURE 4:**
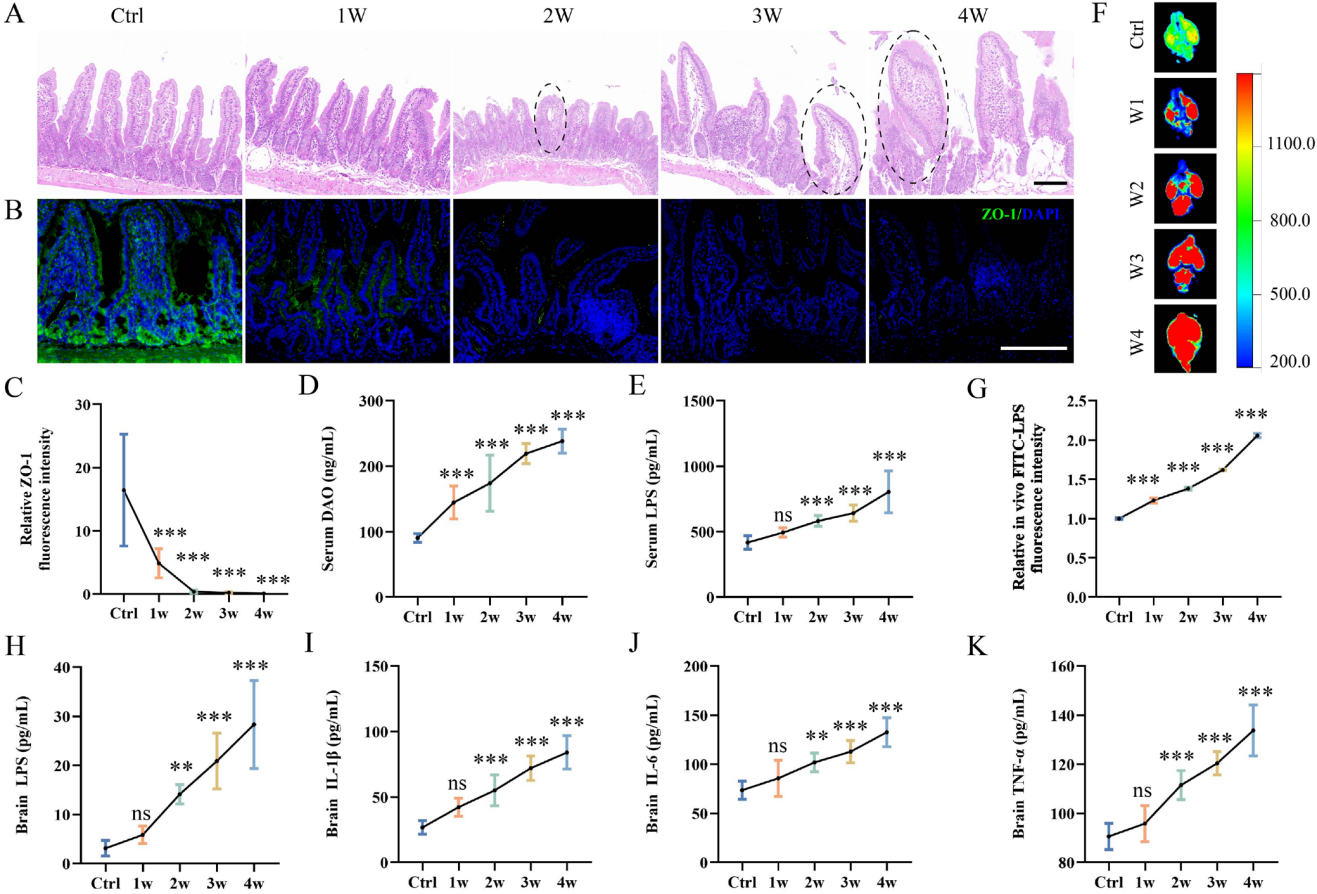
Temporal relationship between intestinal barrier dysfunction and neuroinflammation in chronic alcohol‐exposed mice. (A) Representative H&E staining of jejunal tissue at indicated time points, Ctrl, control group; 1–4 W, oral gavage of ethanol for 1/2/3/4 weeks. Dashed circles indicate swollen and damaged intestinal villi. Scale bar = 100 μm. (B) Representative immunofluorescence images of tight junction protein ZO‐1 (green) in jejunal epithelium (×400). Nuclei were counterstained with DAPI (blue). Scale bar = 200 μm. (C) Quantification of relative ZO‐1 fluorescence intensity. (D) Serum diamine oxidase (DAO) levels. (E) Serum lipopolysaccharide (LPS) levels. (F) In vivo intestinal permeability assessed by FITC‐LPS. (G) Quantification of relative FITC‐LPS fluorescence intensity. (H) LPS levels in brain tissue homogenates. (I–K) Levels of pro‐inflammatory cytokines IL‐1β (I), IL‐6 (J), and TNF‐α (K) in brain tissue. **p < 0.01; ***p < 0.001; ns, *p* > 0.05 vs. W0. Data in (C–E, G–K) were analyzed by one‐way ANOVA followed by Bonferroni post hoc comparisons. *n* = 8 per time point for (C–E, G–K). All data are presented as mean ± SEM.

Consistent with structural damage, functional indicators of intestinal barrier disruption showed early elevation. Serum DAO levels and intestinal permeability were significantly increased from week 1 and continued to rise with prolonged alcohol exposure (*p* < 0.001 vs. W0, Figure 4D,F,G). Serum LPS concentration followed a similar pattern, significantly elevated from week 1 with progressive increases thereafter (*p* < 0.001 vs. W0, Figure 4E).

In striking contrast, central inflammatory markers exhibited a delayed temporal profile. Brain tissue LPS levels showed no significant change at week 1 (*p* > 0.05 vs. W0, Figure 4H), began to increase significantly at week 2, further increased at week 3, and reached peak elevation at week 4 (*p* < 0.001 vs. W0, Figure 4H). Similarly, hippocampal pro‐inflammatory cytokines IL‐1β, IL‐6, and TNF‐α showed no significant elevation at week 1, first became significantly increased at week 2 (*p* < 0.01), progressively rose at week 3 (*p* < 0.001), and attained maximal levels at week 4 (*p* < 0.001 vs. W0, Figure 4I–K).

### 3.5 EA at ST36 Attenuates Alcohol‐Induced Intestinal Barrier Injury and Central Neuroinflammation in Mice

The pCLE revealed heterogeneous fluorescence staining on jejunal mucosal surfaces in the Alc, indicating impaired mucosal integrity accompanied by prominent villus cell desquamation and necrosis (Figure 5A). H&E staining further confirmed these pathological changes, demonstrating jejunal villus atrophy, epithelial damage, lamina propria exposure, and inflammatory cell infiltration in the Alc (Figure 5A). Quantification of relative ZO‐1 fluorescence intensity further verified the reduction of intestinal tight‐junction protein ZO‐1 in the Alc group (*p* < 0.05, Figure 5A,C). ST36 electroacupuncture intervention significantly ameliorated these intestinal pathological injuries, restoring mucosal architecture and reducing cellular damage. Consistent with the observed intestinal barrier damage, serum and brain tissue LPS concentrations were significantly elevated in the Alc compared to the Ctrl. EA intervention markedly reduced LPS levels in both serum and brain tissue (*p* < 0.05 and *p* < 0.001, respectively; Figure 5D,E), thereby alleviating alcohol‐induced endotoxemia and its central nervous system penetration.

**FIGURE 5:**
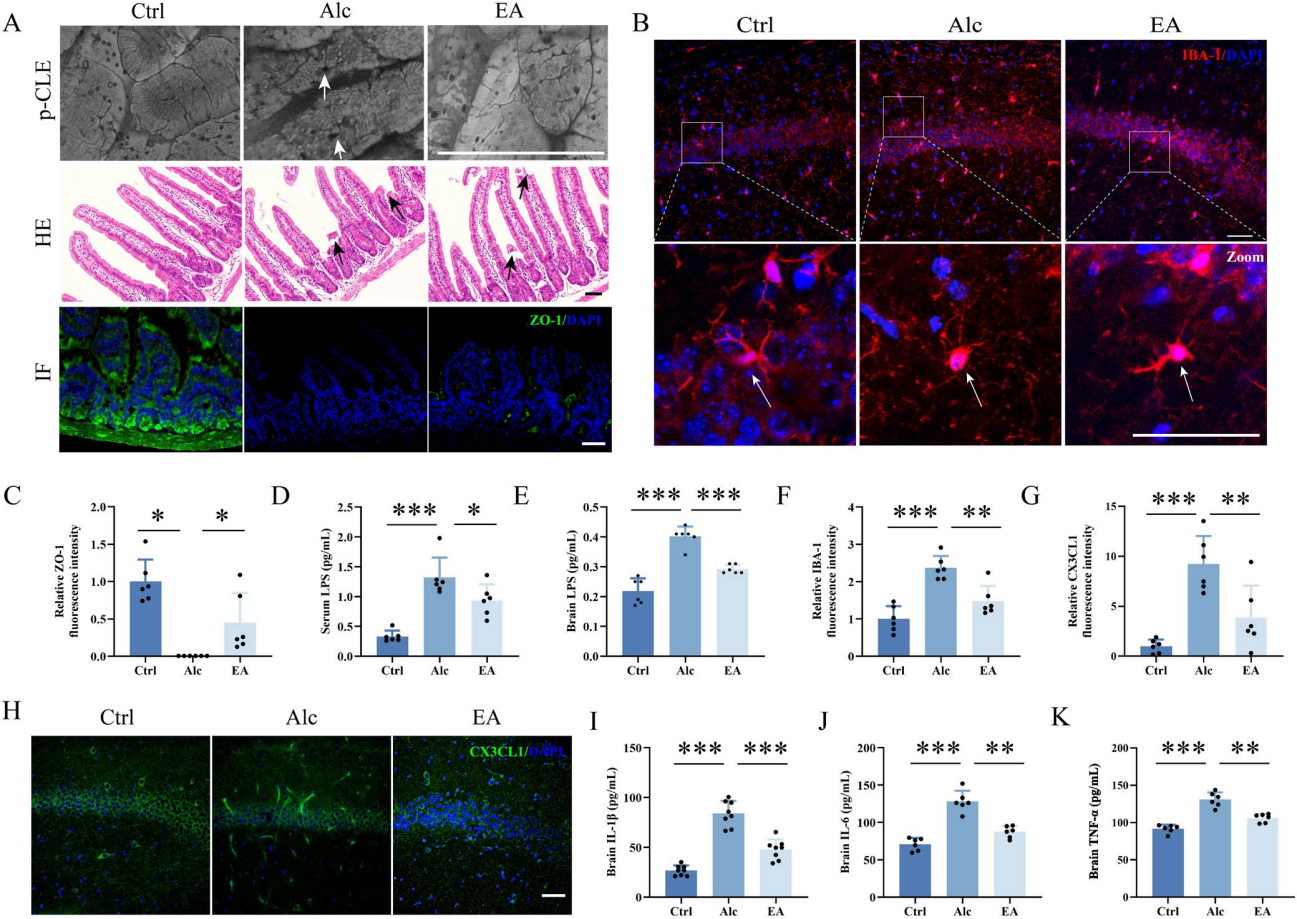
ST36 electroacupuncture restores intestinal barrier integrity and suppresses neuroinflammation. (A) Upper panels: Representative probe‐based confocal laser endomicroscopy (pCLE) images of jejunal mucosa (×1000). White arrows indicate uneven fluorescence due to cell shedding and necrosis. Middle panels: Representative H&E staining of jejunal tissue. Black arrows indicate disrupted intestinal villi. Lower panels: Representative immunofluorescence images of ZO‐1 (green) in jejunal epithelium (×400). Nuclei stained with DAPI (blue). Scale bar = 200 μm. Ctrl, control group; Alc, alcohol‐fed group; EA, alcohol‐fed group treated with EA at ST36. (B) Representative immunofluorescence images of IBA‐1 (red), a microglial marker, in the hippocampus. Arrows indicate activated microglia with amoeboid morphology. Nuclei stained with DAPI (blue). Scale bar = 50 μm. (C) Quantification of relative ZO‐1 fluorescence intensity. (D) Serum LPS levels. (E) Brain tissue LPS levels. (F) Quantification of relative IBA‐1 fluorescence intensity in the hippocampus. (G) Quantification of relative CX3CL1 fluorescence intensity in the hippocampus. (H) Representative immunofluorescence images of CX3CL1 (green) in the hippocampus. Nuclei stained with DAPI (blue). Scale bar = 50 μm. (I–K) Levels of pro‐inflammatory cytokines IL‐1β (I), IL‐6 (J), and TNF‐α (K) in brain tissue homogenates. *p < 0.05, **p < 0.01, ***p < 0.001. Data in (C–G, I–K) were analyzed by one‐way ANOVA followed by Bonferroni post hoc comparisons. *n* = 6 per group. All data are presented as mean ± SEM.

Immunofluorescence staining revealed robust microglial activation in the hippocampus of the Alc, with significantly increased IBA‐1 expression, enlarged cell bodies, and reduced dendritic branching—characteristic features of an activated amoeboid phenotype (Figure 5B,F). EA treatment significantly reversed IBA‐1 upregulation and restored microglial morphology to a ramified, resting state. Furthermore, CX3CL1 expression, a chemokine involved in neuron–microglia communication, was markedly elevated in the hippocampus of the Alc and significantly decreased after EA intervention (*p* < 0.05, Figure 5G,H). Consistent with the microglial activation status, the levels of pro‐inflammatory cytokines IL‐1β, IL‐6, and TNF‐α in brain tissue homogenates were significantly increased in the Alc group, while EA intervention effectively suppressed the overproduction of these pro‐inflammatory factors (*p* < 0.001, *p* < 0.01, and *p* < 0.01, respectively; Figure 5I–K).

### 3.6 EA at ST36 Activates DMV to Ameliorate Intestinal Barrier Injury and Central Neuroinflammation in Chronic Alcohol‐Exposed Mice

In the MWM, the EA + i.DMV and EA + LPS groups exhibited longer escape latencies during the place navigation test (*p* < 0.001 vs. EA, Figure 6B), increased total distance traveled to reach the platform (*p* < 0.001 vs. EA, Figure 6C), fewer platform crossings (*p* < 0.001 vs. EA, Figure 6D), and reduced time spent in the target quadrant (*p* < 0.001 vs. EA, Figure 6E) compared to the EA group. Representative swimming trajectories further illustrated these deficits (Figure 6A). In the OFT, both intervention groups showed decreased total distance traveled (*p* < 0.001 vs. EA, Figure 6G) and reduced time in the center zone (*p* < 0.001 vs. EA, Figure 6H), consistent with reversal of the anxiolytic effects induced by EA (representative trajectories in Figure 6F). Similarly, in the EPM, the mice in EA + i.DMV and EA + LPS groups spent more time in the closed arms (*p* < 0.001 vs. EA, Figure 6J) and made fewer entries into the open arms (*p* < 0.001 vs. EA, Figure 6K), with representative trajectories shown in Figure 6I.

**FIGURE 6:**
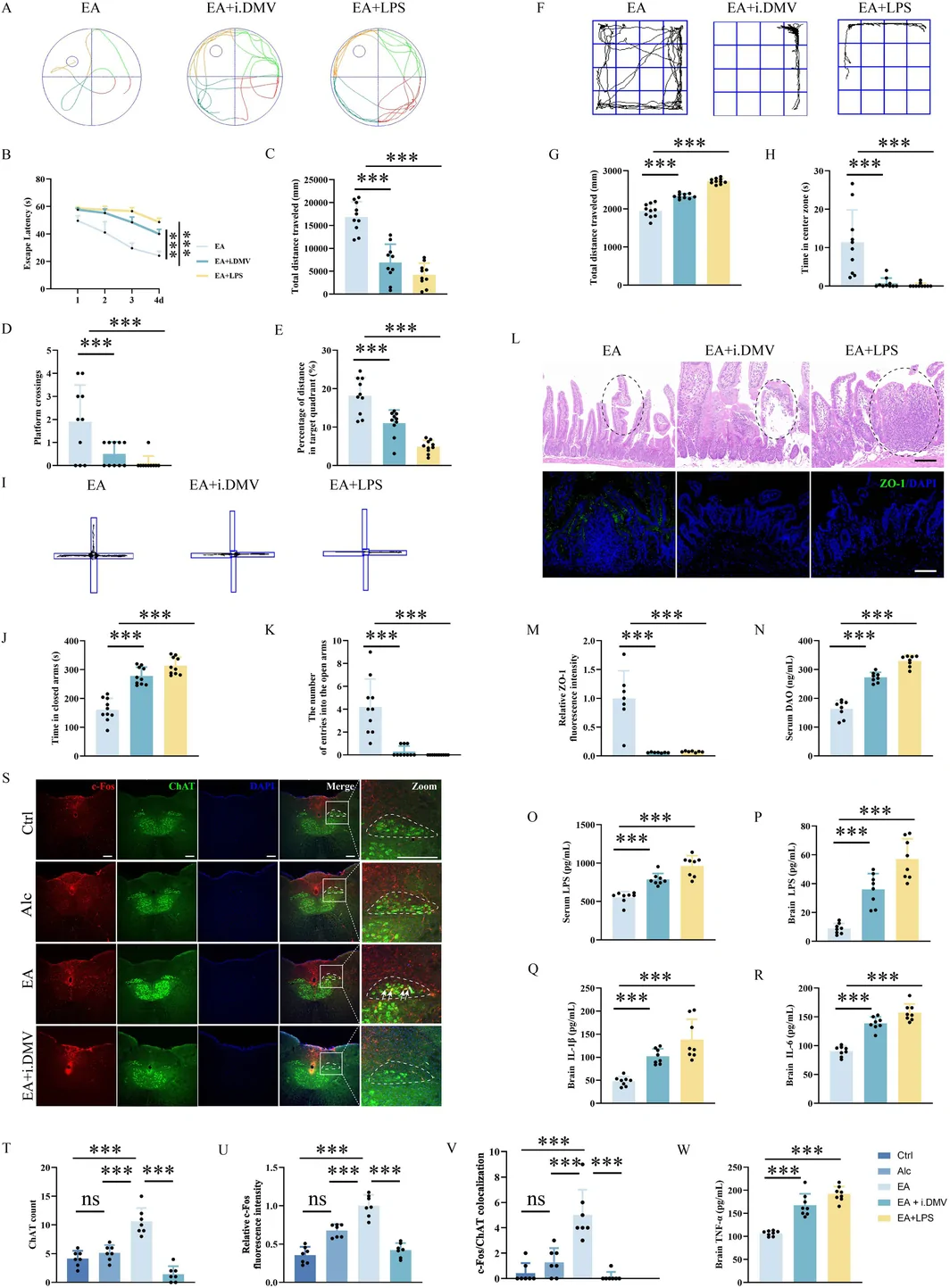
EA at ST36 confers protection via DMV cholinergic neuron activation, and this protection is abrogated by both DMV inhibition and exogenous LPS. (A) Representative swimming trajectories during the MWM spatial probe test. EA + i. DMV, alcohol‐fed mice receiving EA combined with ipsilateral DMV chemical inhibition (muscimol); EA + LPS, alcohol‐fed mice receiving EA combined with intraperitoneal injection of low‐dose LPS. (B) Escape latency during the place navigation test. (C) Total distance traveled to reach the platform on the final test day. (D) Number of platform crossings. (E) Percentage of time spent in the target quadrant. (F) Representative movement trajectories in the OFT. (G) Total distance traveled in the OFT. (H) Time spent in the center zone of the OFT. (I) Representative movement trajectories in the EPM. (J) Time spent in the closed arms of the EPM. (K) Number of entries into the open arms of the EPM. (L) Upper panels: Representative H&E staining of jejunal tissue. Lower panels: Representative immunofluorescence images of ZO‐1 (green) in jejunal epithelium. Nuclei stained with DAPI (blue). Scale bar = 200 μm. (M) Quantification of relative ZO‐1 fluorescence intensity. (N) Serum DAO levels. (O) Serum LPS levels. (P, Q, R) Brain LPS, IL‐1β, and IL‐6 levels. (S) Immunofluorescence co‐staining of ChAT (green) and c‐Fos (red) in the DMV. Arrows indicate ChAT^+^/c‐Fos^+^ double‐positive neurons. Scale bar = 200 μm. (T) Number of ChAT^+^ cholinergic neurons in the DMV. (U) Quantification of relative c‐Fos fluorescence intensity in the DMV. (V) Number of ChAT^+^ neurons co‐expressing c‐Fos. (W) Brain TNF‐α levels. ***p < 0.001; ns, *p* > 0.05. Data were analyzed by one‐way ANOVA, followed by Bonferroni post hoc comparisons. *n* = 8 per group. All data are presented as mean ± SEM.

H&E staining showed that the EA group maintained relatively intact intestinal villus structure, whereas villus swelling and structural disruption were markedly aggravated in the EA + i.DMV and EA + LPS groups (Figure 6L, upper panels). Immunofluorescence staining confirmed robust ZO‐1 expression in the EA group, while ZO‐1 fluorescence was nearly absent in both intervention groups (*p* < 0.001 vs. EA, Figure 6L, lower panels; quantification in Figure 6M). Consistent with these morphological changes, serum DAO and LPS levels were significantly higher in the EA + i.DMV and EA + LPS groups than in the EA group (all *p* < 0.001, Figure 6N,O). Correspondingly, brain tissue LPS levels and pro‐inflammatory cytokines (IL‐1β, IL‐6, TNF‐α) were significantly higher in the EA + i.DMV and EA + LPS groups than in the EA group (all *p* < 0.001, Figure 6P–R,W).

Immunofluorescence double staining for c‐Fos and ChAT was performed to assess the activation of cholinergic neurons in the DMV. Compared with the Ctrl, the number of ChAT^+^ neurons, the relative fluorescence intensity of c‐Fos, and the number of ChAT^+^/c‐Fos^+^ double‐positive neurons were not significantly changed in the Alc (*p* > 0.05, Figure 6T–V). In contrast, EA treatment markedly increased these three parameters compared with the Alc (all *p* < 0.001, Figure 6T–V). Importantly, DMV lesion (EA + i.DMV group) completely abolished the EA‐induced increases in ChAT^+^ neuron number, c‐Fos intensity, and ChAT^+^/c‐Fos^+^ co‐labeling (all *p* < 0.001 vs. EA, Figure 6T–V), demonstrating that activation of DMV cholinergic neurons is required for the protective effects of EA. Representative images are shown in Figure 6S.

## 4 Discussion

Alcohol‐related cognitive impairment (ARCI) is a severe neurocognitive complication of chronic alcohol use disorder [32] and exhibits an extremely high incidence among patients with alcohol abuse or dependence [33], significantly exacerbating long‐term disability and reducing quality of life [34]. Accumulating evidence indicates that intestinal barrier damage plays a key driving role in the pathophysiology of alcohol use disorder. Specifically, chronic alcohol exposure directly damages small intestinal villi—critical components of both mechanical and chemical barriers [35]—manifesting as villus shortening, fusion, or shedding [36], along with disruption of epithelial tight junctions and reduced mucus secretion [37]. These changes impair intestinal barrier integrity, resulting in a “leaky gut” [38] that facilitates translocation of gut‐derived LPS into the systemic circulation [39]. The resulting endotoxemia activates systemic inflammatory signals, which subsequently trigger a neuroinflammatory cascade in the CNS via gut‐brain axis pathways, ultimately driving ARCI onset and progression [40].

LPS induces neuroinflammation primarily by activating microglia, the brain's resident immune cells that are crucial for CNS homeostasis [41]. Under physiological conditions, microglia exert neuroprotective effects [42]; however, excessive activation transforms them into an amoeboid phenotype, releasing pro‐inflammatory cytokines such as IL‐1β and TNF‐α [43] alongside chemokines such as CX3CL1 [44]. Soluble CX3CL1 recruits immune cells and activates microglia via CX3CR1 binding, further amplifying neuroinflammation and exacerbating tissue damage [45]. The hippocampus, a key region for learning, memory, and emotional regulation, is particularly vulnerable to such neuroinflammation [44], with synaptic damage and impaired neurogenesis underlying the cognitive deficits associated with chronic alcohol abuse.

In this study, chronic alcohol‐exposed mice recapitulated the main features of ARCI, including progressive nutritional decline, anxiety‐like behaviors (OFT, EPM), and impaired spatial learning/memory (MWM). These behavioral deficits were accompanied by intestinal barrier disruption (villus damage, reduced ZO‐1 expression), elevated LPS in circulation and brain, hippocampal microglial activation (increased IBA‐1, amoeboid morphology), and upregulated IL‐1β and CX3CL1. Critically, time‐course analysis revealed that intestinal barrier injury and endotoxemia preceded neuroinflammation by approximately one week, supporting the causal role of gut‐derived LPS in triggering CNS inflammatory responses. EA at ST36 intervention significantly attenuated all these pathological changes, mitigating intestinal damage, restoring barrier integrity, reducing LPS leakage, inhibiting microglial activation and neuroinflammation, and ultimately ameliorating cognitive and emotional deficits.

A key mechanistic insight relates to vagal involvement in mediating EA effects [18]. The vagus nerve, a core regulator of the gut‐brain axis, exerts systemic anti‐inflammatory effects through the cholinergic anti‐inflammatory pathway (CAP). We observed increased activation of ChAT^+^/c‐Fos^+^ neurons in the DMV after EA, reflecting enhanced vagal efferent signaling, consistent with previous studies showing that EA stimulation alleviates intestinal inflammation through vagal cholinergic mechanisms [24]. Importantly, DMV lesion abolished EA's protective effects on intestinal barrier integrity, neuroinflammation, and behavior, confirming that vagal integrity is required. Furthermore, exogenous LPS challenge completely abrogated EA‐induced protection—when LPS was administered intraperitoneally, bypassing the intestinal barrier, EA failed to improve cognitive function, intestinal barrier damage, or neuroinflammation. This critical finding demonstrates that in mice exposed to chronic alcohol, the brain‐protective effect of EA may mainly be achieved by improving the intestinal barrier function to block the leakage of LPS, rather than by directly inhibiting LPS‐induced inflammatory cascades in the brain.

Our findings support EA at ST36 as a promising non‐pharmacological intervention for ARCI. Given the current limitations of pharmacotherapies for this condition, acupuncture targeting the gut‐brain axis holds significant clinical potential. However, the precise molecular mechanisms by which ST36 stimulation activates vagal pathways and promotes intestinal repair remain to be fully elucidated. Future studies integrating multi‐omics approaches (e.g., microbiomics, metabolomics) and neural circuit tracing will provide deeper mechanistic insights and guide clinical trial design.

## 5 Conclusion

EA at ST36 ameliorates anxiety‐like behaviors and cognitive deficits in a mouse model of ARCI through restoration of intestinal barrier integrity. EA activates DMV cholinergic neurons, upregulates ZO‐1 expression to prevent gut‐derived LPS translocation, and subsequently suppresses hippocampal microglial activation and pro‐inflammatory cytokine release. Time‐course analysis confirmed that intestinal barrier injury precedes neuroinflammation; DMV lesion and exogenous LPS challenge demonstrated that EA acts via a vagus‐dependent mechanism upstream of LPS. This study identifies the LPS–gut–vagus axis as a key therapeutic target and supports EA at ST36 as a non‐pharmacological intervention for early ARCI.

## Author Contributions

X.S. conceived and devised the experiments; Y.L., J.L., Y.W., and S.W. executed them; J.L. and S.J. collected data and information. G.W. verified the references and polished the language. Y.L., W.X., and X.S. tabulated the data and authored the manuscript. All contributors reviewed and approved the final version for publication.

## Funding

This work was supported by the National Natural Science Foundation of China (82274650); the Innovation Capacity Enhancement Special Project of the Institute of Acupuncture and Moxibustion, China Academy of Chinese Medical Sciences, CIZIA2024‐003.

## Ethics Statement

The animal experiments were approved by the Medical Ethics committee of Xiyuan Hospital of China Academy of Chinese Medical Sciences (No. 2025XLC037‐2).

## Conflicts of Interest

The authors declare no conflicts of interest.

## Supporting information


**Table S1:** Antibodies used for immunofluorescence.

## Data Availability

The data that support the findings of this study are available from the corresponding author upon reasonable request.
