# Supplementary material for: Electroacupuncture Ameliorates Alcohol‐Related Cognitive Impairment by Modulating the Gut‐Brain Axis to Alleviate LPS Driven Neuroinflammation
Source: CNS Neurosci Ther. 2026 Oct 16;32(10):e71213. doi: 10.1002/cns.71213 (PMC13674736; doi:10.1002/cns.71213)
Supplement: Supplementary file 1 [file CNS-32-e71213-s001.docx]

**Supplementary Table 1.** Antibodies used for immunofluorescence.

| Antibody | Company | Catalog number | Dilution |
| --- | --- | --- | --- |
| Anti-IBA-1 | FUJIFILM Wako Pure  Chemical Corporation | 013-26471 | 1:200 |
| Anti CX3CL1 | Abmart | TD12376 | 1:500 |
| Anti-c-Fos | Abcam | **AB208942** | 1:1000 |
| Anti-choline  acetyltransferase | Abcam | **AB178850** | 1:1000 |
| ZO-1 | Santa Cruz | **SC33725** | 1:100 |
| Alexa Fluor 488-conjugated goat anti-rabbit IgG H&L | MedChemExpress | HY-P8002 | 1:500 |
| Alexa Fluor 647-cojugated goat anti-mouse IgG H&L | Abcam | **AB150115** | 1:500 |
